# Revisiting the Complications of Orthodontic Miniscrew

**DOI:** 10.1155/2022/8720412

**Published:** 2022-08-01

**Authors:** Van Mai Truong, Soyeon Kim, Jaeheon Kim, Joo Won Lee, Young-Seok Park

**Affiliations:** ^1^Department of Oral Anatomy and Dental Research Institute, School of Dentistry, Seoul National University, Seoul 03080, Republic of Korea; ^2^Head of Center for Future Dentistry, School of Dentistry, Seoul National University, Seoul 03080, Republic of Korea

## Abstract

Miniscrew has been used widely as an effective orthodontic anchorage with reliable stationary quality, ease of insertion and removal techniques, immediate or early loading, flexibility in site insertion, less trauma, minimal patient cooperation, and lower price. Nonetheless, it is not free of complications, and they could impact not only the miniscrew success rate but also patients' oral health. In this article, literature was searched and reviewed electronically as well as manually to evaluate the complications of orthodontic miniscrew. The selected articles are analyzed and subcategorized into complications during and after insertion, under loading, and during and after removal along with treatment if needed according to the time. In addition, the noteworthy associated factors such as the insertion and removal procedures, characteristics of both regional and local anatomic structures, and features of the miniscrew itself that play a significant role in the performance of miniscrews are also discussed based on literature evidence. Clinicians should notice these complications and their related factors to make a proper treatment plan with better outcomes.

## 1. Introduction

Orthodontic anchorage is a prerequisite for the success of orthodontic treatment. Various types of anchorages are available, composing onplants, palatal plates, miniplates, and miniscrews [1]. Among them, miniscrews have been used more widely for orthodontic anchorage reinforcement due to good stationary quality, various insertion sites, simple placement or removal procedures, light tissue invasion, immediate or early loading allowance, minimal patient compliance, and low cost [2–4]. Miniscrews have been proved to provide reliable anchorage and placed in numerous clinical applications such as deep bite correction, space closer, midline correction, extrusion, intrusion, distalization, mesialization, and en-masse retraction [2] with high success rate [5]; in addition, the uses of miniscrew have widened the scope of nonsurgical orthodontic therapy [6]. Moreover, it was shown that miniscrews could facilitate more favorable outcomes compared with conventional methods [7, 8]. Nonetheless, complications could occur not only during and after insertion but also under loading, during, and after removal. It is necessary for clinicians to comprehensively understand its complications and related factors to minimize the failure rate.

## 2. Complications during Insertion

### 2.1. Root Contact

The insertion of orthodontic miniscrews in interradicular regions could lead to iatrogenic root damage. Among the complications, its outcome could be considered the most serious for the patient's dental health [9]. Potential complications of root injury include loss of tooth pulp sensibility, root resorption, root fracture, osteosclerosis, and dentoalveolar ankylosis [10, 11].

The periradicular lesion as a consequence of root proximity could be successfully treated with endodontic treatment and apical surgery with mineral trioxide aggregate [12–14]. Although it was possible to be repaired successfully, the need for particular attention should be highlighted during miniscrew placement to decrease the risk of root damage. Increased failure rates of miniscrew placement were detected among those contacting adjacent roots [15]. It was also found that root damage was a crucial risk factor for miniscrew failure [16].

A perforated root has a capability of spontaneous repairing and regenerating after immediate removal of the offending miniscrew and additional stimulation for an adequate period, and orthodontic therapy could be finished without unfavorable symptoms [17–19]. The injured root could be monitored for possible revitalization and regeneration rather than performing endodontic therapy instantaneously [19]. Nevertheless, this could occur only when root damage caused by miniscrew placement is limited to the cementum or the dentin without inflammatory infiltrate or pulpal injury [20]. Cases that miniscrews invaded the pulp were less feasible to archive absolute repair of the periodontal tissues [9]. On the other hand, it was reported that insertion of the miniscrew into the periodontal ligament even less than 1 mm could cause external root resorption [21].

Insertion torque with root contact was proved to be higher than those without and in agreement with many studies [22]. One study showed that the average placement torques in contact cases were twice higher than those in noncontact cases [20]. For that reason, increased resistance during miniscrew insertion was recognized as an indicator of root contact [20]. Nonetheless, bone density might be diverse among individuals and placement locations. Besides, even under topical anesthesia, when the miniscrew started to contact the periodontal ligament, increased sensation could be felt by the patient [23, 24]. Once doubtful symptoms of root proximity are noticed, taking periapical radiography and cone-beam computed tomography is recommended to approve and assess the root status [19].

For the anterior region, the area with the greatest amount of interradicular bone for miniscrew placement was between the lateral incisor and the first premolar [25]. For the posterior area, the region between the second premolar and the second molar was suggested to be the safest zone [26]. In the maxilla, the best option was from the second premolar to the first molar, from 6 to 8 mm from the cervical line [27]. In the mandible, the most favorable zone was from the first molar to the second molar, below 5 mm from the cervical line [27].

It is important to make a careful plan for miniscrew insertion to minimize the potential of root damage [18]. The application of surgical guides, fabricated using cone-beam computed tomography (CBCT) images, could be considered a technique for more accurate orthodontic miniscrew placement adjacent to important anatomic structures [28–31].

### 2.2. Perforation of Maxillary Sinus and Nasal Cavity Floor

During orthodontic miniscrew installation, perforation into the nasal cavities and maxillary sinuses has been reported [32–35]. Infrazygomatic crest anchorage has been applied successfully for anterior retraction, space closure, posterior intrusion, and molar and maxillary dental arch distalization [32]. This region may be particularly amenable to miniscrew insertion due to the two cortical layers that will ensure primary stability if a miniscrew with proper length could be fixed bicortically [34]. Bicortical miniscrews provide higher anchorage resistance, lower cortical bone stress, and better stability in comparison with monocortical miniscrews [3].

In the palate, distance to the nasal cavity and maxillary sinus was greatest in the region mesial to the first premolar and then the distance started to decrease significantly [36]. In the buccal area, perpendicular insertion was safe with minimal risk of sinus or nasal cavity injury, while oblique placement increased the possibility of sinus and Schneiderian membrane penetration [36–38].

The response of the maxillary sinus to perforation by dental implants has been assessed; a simple perforation smaller than 2 mm may heal spontaneously without complications [10]. However, in the case of perforation by orthodontic miniscrews, this might not apply, since the implant size, loading pattern, surrounding bone characteristics, and blood flow may be different compared to dental implants [32]. Even though in some studies miniscrew removal and interruption of orthodontic therapy after perforation were not operated without complications, it may be a risk factor for miniscrew failure [33]. A sinus perforation at a depth not exceeding 1.5 mm did not seem to affect miniscrew anchorage [33]. However, a study reported that nasal floor perforation caused oronasal fistula development during wound healing and surgery had to be performed to close it after miniscrew removal [35].

Therefore, clinicians should consider primary stability with sinus health status at the same time. Infrazygomatic crest miniscrew anchorage was recommended to be bicortical fixed with penetration depth limit within 1 mm [32]. To achieve it, the infrazygomatic crest region should be fully analyzed using CBCT, considering individual differences; virtual miniscrew insertion in the CBCT scans was also advisable for deciding miniscrew size and placement angulation [32].

### 2.3. Cortical Bone Damage

Extensive osseous microdamage during insertion of orthodontic miniscrew may reduce the stability of immediately loaded miniscrews due to the bone remodeling processes initiated by microdamage [39]. Large diameter miniscrews and overtightening through deep insertion might lead to more significant microdamage to the cortical bone [39, 40]. Nevertheless, miniscrews with too small diameters could raise the potential of miniscrew fracture during placement and mobility of the miniscrews when orthodontic force is applied due to the low resistance to removal torque. Miniscrews with diameters of 1.5 or 1.6 mm were recommended as a compromise between the physical properties of miniscrews and microdamage in the cortical bone [40].

Pilot drilling might be an effective solution to reduce microdamage during insertion [41, 42]. After pilot drilling, both the miniscrew diameter and the insertion site (mandible vs maxilla) had no significant effect on the amount of microdamage around the miniscrew [41]. Moreover, to prevent excessive microdamage, large diameter and cylindrical miniscrews should be avoided [42].

Regarding the insertion technique, more serious microdamage in the cortical bones was observed in both maxilla and mandible by the self-drilling placement technique in comparison with the pre-drilling (self-tapping) one [43]. Besides, the cortical bone thickness was shown to have a significant influence on the amount of microdamage created instantly after placement. There was a statistically significant positive association between cortical bone thickness and the amount of microdamage [44]. It was suggested that practicians should take into account the thickness of cortical bone at the placement site, because reducing cortical bone thickness will likely decrease the amount of microdamage created during insertion [44].

### 2.4. Miniscrew Fracture

Increased torque placement could cause miniscrew bending or fracture that not only affects the miniscrew stability but may also requires surgical intervention. Miniscrew fracture has been reported and caused a sinus tract, and the fractured tips had to be removed surgically [13, 45].

Miniscrews from different manufacturers have different designs and morphology; outside and internal diameters, the ratio of these two diameters, and milling in miniscrew apical region were the factors that decide the fracture torque resistance [46]. Fracture most likely happens in the cervical part of the miniscrew because of mechanical stress focusing at this point [45]. Stress distribution on the miniscrew surfaces and the adjacent bone in force application was proved to be related to insertion depth and angulation [47, 48]. Miniscrew inclined insertion with upward traction was recommended to be the safest option to prevent miniscrew failure and fracture [47].

This complication could be prevented or limited by choosing the appropriate placement torque with a suggested range from 5 to 10 N.cm [49, 50]. In addition, for self-drilling miniscrew, a pilot hole should be applied beforehand to prevent excessive torque [50].

## 3. Complications after Insertion

Installation of miniscrews may cause pain and discomfort [35, 51–56]. Pain intensity and discomfort were not greater than other orthodontic procedures, and some authors reported that patients preferred miniscrews to tooth extraction [51–53]. Therefore, patients were willing to adopt the new orthodontic treatment, and this did not negatively affect the final general satisfaction with the treatment [52, 55]. However, insertion with extra-alveolar bone miniscrews and flap surgery was shown to cause more pain than that of the smaller miniscrew and nonflap surgery [56, 57].

Prolongation of pain most likely happened in the anterior teeth [35]. This might be caused by the interference fit of the palatal miniscrew created after placement [35]. One case in which pain lasted until the miniscrew got loose was reported; this might be triggered by compressing or contacting the incisal nerve [35]. Although permanent nerve injury after miniscrew installation has not been described in the literature, precautions should be taken to avoid nerve involvement.

Secondary bleeding after miniscrew insertion may also happen. Prolonged bleeding could be stopped by compression. If this method does not work, clinicians can constrict the bleeding vessel or use electrocautery to stop bleeding [35].

## 4. Complications under Loading

### 4.1. Stationary Anchorage Failure

Many risk factors could affect the stability of miniscrew: patient-related (age and sex), miniscrew-related (diameter, length, and design), location-related (thickness of cortical bone, density of bone, thickness and type of soft tissue, and insertion site), and clinical procedure-related (pre-drilling/self-drilling, pilot hole, and method of loading). The affections of these factors were significantly different between studies.

Among risk factors from patients, the association between miniscrew failure and age was not consistent. Some studies showed no relationship between age and failure [58–60]; whereas others found that age could affect the miniscrew stability since there was poorer quality and higher bone turnover rate in growing patients compared to adults, affecting optimal mechanical miniscrew stability in adolescents [61–65]. Therefore, more attention should be taken to the miniscrew placement in younger patients. The affection of sex was also found to be controversial. While some studies reported that there were no statistical differences [58–60, 65], others reported that males had a higher success rate due to higher bone density [59, 62].

Regarding miniscrew characteristics, an increase in miniscrew diameter and length could reinforce the initial stability [65–67]. Nonetheless, the proximity of anatomical structures should be considered. A study reported that miniscrews of 1.2-mm diameter and at least 8-mm length were favorable for the reason that they were stable and limited the probability of root injury [68]. In another study, higher success rates were also found with the same length of miniscrews (≥8 mm), but slightly higher diameter (>1.4 mm) [65]. Miniscrew stability may also vary with its design, and a study suggested that a conical miniscrew design would provide greater primary stability than a cylindrical miniscrew type [69].

The success of orthodontic miniscrews may be affected by various location-related factors. Miniscrew stability was positively associated with the cortical bone thickness of the insertion site [66, 70, 71]. Motoyoshi et al. reported that a cortical bone thickness threshold of 1 mm increased the miniscrew success rate [72]. Bone density may also play a role in miniscrew failure; with the same length of miniscrew, good anchorage resistance was obtained only in bone with optimum density [73]. However, another study proved that there were no established associations between bone density and miniscrew success rate [74, 75]. In addition, one study showed that miniscrew stability was associated linearly with insertion depth, extrabony miniscrew length may also be a determinant of miniscrew stability, and it was suggested that insertion sites should be selected so that mucosa there is as thin as possible [76]. This was in an agreement with another study that the thickness of the soft tissues was an important factor in the success of orthodontic miniscrew; due to soft tissue thickness variation, clinicians should consider before selecting a miniscrew [77]. Moreover, placement in attached gingiva seemed to be more favorable to achieving higher success of miniscrew compared with insertion in movable mucosa [67, 78]. Maxilla placement of miniscrews was more successful than mandible [61, 65, 78, 79] due to more keratinized tissue, less challenging surgical technique, greater vascularization of the maxilla or greater bone overheating during drilling [78], and irritation during chewing of the mandibular [79]. Meanwhile, another study concluded that significant differences in the success rates among receptor sites were found only with pre-drilling miniscrews; it was suggested that the insertion procedure might play a certain role in the success rate of miniscrew [80].

Self-drilling miniscrews have been reported to reduce clinical time, bone damage, and patient discomfort compared with pre-drilling miniscrews [81]. A systematic review suggested that there were no differences between the success rates of pre-drilling and self-drilling miniscrews [82]. With pre-drilling miniscrew, a pre-drilled hole is required. The relationship between the diameter of the pilot hole and miniscrew stability was summarized as follows: the larger pilot hole compared to miniscrew diameter is, the lower the primary stability of miniscrew is; the smaller pilot hole compared to miniscrew diameter is, the more potentially miniscrew will fracture [83]. To have proper insertion torque and to prevent miniscrew fracture as well as extreme bone stress, there should be an ideal combination of pre-drilling pilot and miniscrew diameter; it was recommended that the drill diameter should be 0.5 mm smaller than that of miniscrew [84].

Inadequate primary stability might be also caused by overwinding during miniscrew installation. Open flap technique was performed for better vision and to prevent overwinding. Nonetheless, a necrotic mucosa of miniscrew was observed only in cases with flap surgery [35]. In addition, the success rates presented for flap and flapless procedures were not homogeneous among studies [67].

Applying low-level laser was shown to increase the stability of miniscrew and peri-screw bone formation [85]. In addition, a small diameter decortication using Er:YAG laser might produce better primary stability in comparison with using a drill; thus, it could be used as an alternative [86]. However, additional studies should be employed to confirm the results.

There has been controversy regarding the waiting period between miniscrew placement and orthodontic loading. On the other hand, immediate loading was shown to give favorable contact with the adjacent bone and not affect miniscrew anchorage [87–89]. Even though immediate or early loading of miniscrews can be applied, the limit of force at 200 cN was recommended [68]. The direction of the orthodontic force may also affect the primary stability of miniscrews [90]. Dislodgement of miniscrew occurred most frequently in the first 2 months and mostly within the first 4 months [60]. In addition, clinicians' experience and skill also play an important role in the success rate of miniscrew [63, 91].

Unfortunately, the diversity of analyzed factors of miniscrew anchorage may lead to bias; for that reason, homogenous groups of patients are necessary for reliable assessment. The analyzed factors should be treated with caution due to the different methodologies employed in different studies.

### 4.2. Miniscrew Displacement

Although miniscrews have been affirmed to provide good stationary quality, many studies confirmed that there was a remarkable secondary displacement of the miniscrew under orthodontic loading over time [89, 92–95]. However, this displacement did not appear to affect the clinical performance of miniscrews [93, 95].

The amount of movement is clinically considered since there is potential to interfere with vital structures such as foramen, nerves, blood vessels, or dental roots. The safe zone between miniscrew and dental root varied among studies, extending from 1.5 mm to 2 mm for prevention [89, 92]. However, one study reported that the mean secondary dislocation was from 0 to 2.7 mm for entire miniscrews; also, controlled tipping and bodily movements were the most common [93]. For safety, further research should be investigated for predicting the optimal zone.

Bone mineral density rather than cortical bone thickness was the key factor in controlling the primary migration of miniscrew under functional orthodontic loading [94]. Additionally, both pre-drilling and self-drilling miniscrews showed displacement under loading, and the quantity of dislocation was related to the period of loading time without noticeable mobility or loosening [95].

### 4.3. Traumatic Soft Tissue Lesion and Soft Tissue Coverage

Traumatic soft tissue lesions could happen in the form of aphthous ulcerations or canker sores in alveolar, buccal, labial mucosa, or frenulum [10, 96]. However, these injuries are self-limiting and able to heal without further complications. Using healing abutment, wax pellet, and elastic separator over the head of miniscrew, with daily use of chlorhexidine, was performed for ulceration prevention and patient comfort improvement. The appearance of a traumatic lesion was not considered a direct risk factor for the anchorage of miniscrew; however, it may be a sign of more severe soft tissue inflammation [10].

Light-cured temporary filling material was used to cover the head of the miniscrew, and this is a simple method that was recommended for soft tissue trauma prevention [96]. However, using composite resin could make ligating to the miniscrew harder; furthermore, the contact between the composite resin and peri-screw tissue may cause allergy if the patient is sensitive to the components of the material [97]. Thus, an article suggested a modification method by placing an elastomeric separator around the head to maintain some space between the composite resin and the peri-screw tissue [97].

Overgrowth, defined as the partial or complete covering of the miniscrew head by soft tissue, was reported to be the most common complication in one study with no treatment needed. Although it did not bring serious complications but could cause time-consuming annoyance, this complication might be prevented by either decreasing the insertion depth or using miniscrews with longer necks. However, due to primary and long-term anchorage, miniscrews with longer necks may be preferable; besides, it is necessary for patients to maintain good oral hygiene [98].

### 4.4. Peri-screw Inflammation

Inflammation around the miniscrew was reported to occur in the regions of palate, buccal fold, and ascending ramus [45]. Peri-screw inflammation was associated with miniscrew failure [74, 99]. In patients with poor oral hygiene, inflammation can happen even if the placement procedure is operated carefully [45].

Presurgical and postsurgical oral hygiene was considered a critical factor to prevent peri-screw inflammation [45, 64, 100]. Patients need to have thorough oral care education, and professional cleaning may be also necessary for the orally exposed part of the miniscrews [101].

Miniscrews inserted in the buccal surface of the alveolar process and the alveolar mucosa had a greater chance to have inflammation [45, 102]. Control of infection is a fundamental factor to ensure the stability of the miniscrew [102]. Local disinfectants, antiseptic mouthwash, and careful brushing techniques were recommended for this purpose [45, 101]. In more severe cases, antibiotics [101] or miniscrew removal and repositioning in another site [102] may be needed, even though no stability loss was observed.

## 5. Complications during Removal

During removal, miniscrew fracture can happen if the torque is over the limit of the miniscrews [80]. For this reason, controlling the removal torque was recommended [103]. In addition, partial osseointegration surrounding miniscrews could be obtained after insertion, and fracture might not be avoided if it is the result of the strength of osseointegration [80]. Fracture of miniscrew during removal has been reported and had to be retrieved by surgery [35] which may lead to significant bone removal and potential risks for patients [80].

Even though it was reported that miniscrews could be removed with hand-operated drivers while controlling torque, using battery-operated drivers or other units which do not offer torque limitation in reverse mode was not advisable [103]. Besides, it may be a favorable method to use ultrasonic instruments for orthodontic miniscrew removal due to less bone loss and faster bone healing in comparison with using low-speed handpiece rotary instruments [104].

One study showed that applying sandblasting and acid etching for surface roughness did not improve the success rate but increased the removal torque significantly which may raise the risk of miniscrew fracture [105]. Moreover, a miniscrew with smaller diameters and made of ductile titanium alloy may also collaborate to increase the chance of miniscrew fracture during removal [80].

## 6. Complications after Removal

In general, orthodontic miniscrew removal is not considered a traumatic approach. However, after removal, there will be a temporary full-thickness defect through soft tissue and alveolar bone underneath, which is healed by secondary intention [106].

### 6.1. Soft Tissue Scarring

After orthodontic miniscrew removal, detectable soft tissue scarring may develop at a fairly high rate [106, 107]. Even though this scarring was only located at the site of placement and was not considered serious, it might give negative esthetic problems [106]. The scar tissues were excised successfully under local anesthesia, but further studies should be investigated for soft tissue healing improvement and visible scarring prevention [106]. Flat gingiva and buccal interdental gingival insertion are more likely to have scar formation [107]. Proper miniscrew placement torque values may limit the probability of not only negative tissue responses such as scar tissue formation but also micromotion [80].

### 6.2. Bone and Root Resorption

Excess microdamage created during miniscrew insertion may cause bone resorption [108]. In addition, based on the presently available evidence, miniscrew-assisted intrusion is a risk factor for orthodontically induced inflammatory root resorption; however, a variety of related characteristics (such as insertion site, intrusion site, duration, and magnitude of intrusive force) may have influence on the outcome [109]. It was believed that the magnitude of intrusive force was associated directly with the root resorption [110]. Nevertheless, due to methodological inconsistencies, it was challenging to quantitatively assess the results [109]. During this process, the application of photobiomodulation might have a possibility to lower the progression of root resorption, but it may also slightly lower intrusion distance and speed [111].

### 6.3. Alveolar Bone Exostoses

An alveolar bone exostosis is a localized, peripheral bone overgrowth with unknown pathogenesis; but the potential factors could be race, autosomal dominance, hyperfunctional masticatory, and nutrition [112]. Normally, for alveolar bone exostosis, the treatment will not be operated unless its size affects the periodontal tissue or causes pain and discomfort for patients [112]. Alveolar bone exostoses have been reported once in the literature as a complication of orthodontic miniscrew [113]. In this case, resective osseous surgery was performed, and orthodontic treatment was continued after one month without recurrence [113].

## 7. Conclusions

This article has focused on pointing up the complications of miniscrew and their related factors reported through the literature that are summarized in Table 1. It is suggested that clinicians should thoroughly understand the insertion and removal procedures and characteristics of both regional and local anatomic structures as well as features of the miniscrew itself to optimize the success rate. Attention should be given that there may be biases between studies due to the diversity of analyzed factors along with markedly heterogeneous protocols. Future studies are needed to address a standard protocol with homogeneity.

## Figures and Tables

**Table 1:** Complications of miniscrew, their related factors, and treatment.

Complications		Related factors	Treatment
During insertion	Root contact	(i) Interradicular region (ii) Damage location: cementum/dentin/pulp (iii) Insertion torque: increased resistance (iv) Increased sensation by patient	(i) Immediate removal (ii) Endodontic treatment/apical surgery
	Perforation of maxillary sinus and nasal cavity floor	(i) Infrazygomatic crest anchorage (ii) Perforation depth	(i) No treatment (ii) Miniscrew removal (iii) Surgery performed to close the perforation
	Cortical bone damage	(i) Large diameter miniscrew (ii) Overtightening (iii) Pilot drilling and insertion technique (self-drilling/pre-drilling) (iv) Cortical bone thickness	(i) No treatment
	Miniscrew fracture	(i) Designs and morphology of miniscrew (ii) Placement torque (iii) Insertion depth and angulation (iv) Pilot drilling and insertion technique	(i) Surgical intervention to remove the fractured tip
After insertion	Pain and discomfort	(i) Size of miniscrew (ii) Flap/nonflap surgery	(i) No treatment
	Secondary bleeding	(i) Unknown	(i) Compression (ii) Constrict the bleeding vessel (iii) Use electrocautery
Under loading	Stationary anchorage failure	(i) Age, sex (ii) Diameter, length, design of miniscrew (iii) Thickness of cortical bone, density of bone, thickness and type of soft tissue, insertion site (iv) Pre-drilling/self-drilling, pilot hole, method of loading	(i) Miniscrew removal (ii) Repositioning
	Miniscrew displacement	(i) Bone density and cortical bone thickness (ii) Period of loading time	(i) No treatment unless it affects the vital structures
	Traumatic soft tissue lesion	(i) Head of miniscrew	(i) No treatment
	Soft tissue coverage	(i) Insertion depth (ii) Length of miniscrew neck	(i) No treatment
	Peri-screw inflammation	(i) Insertion region (ii) Oral hygiene	(i) Control of infection (ii) Miniscrew removal (iii) Repositioning
During removal	Miniscrew fracture	(i) Removal torque (ii) Instrument used for removal (iii) Partial osseointegration (iv) Diameter and surface roughness of miniscrew	(i) Surgical intervention to remove the fractured part
After removal	Soft tissue scarring	(i) Flat gingiva (ii) Buccal interdental insertion	(i) Scar removal by excising
	Bone and root resorption	(i) Bone microdamage (ii) Miniscrew-assisted intrusion (iii) Intrusive force	(i) No treatment
	Alveolar bone exostoses	(i) Unknown	(i) Resective osseous surgery

## Data Availability

The data was obtained from PubMed database.
